# Using the precaution adoption process model and the health belief model to understand radon testing and mitigation: a pre-post quasi-experimental study

**DOI:** 10.1186/s12889-023-15752-2

**Published:** 2023-05-19

**Authors:** Allison Maier, Erin Hayes, Lisa Munday

**Affiliations:** Kingston, Frontenac, and Lennox & Addington Public Health, 221 Portsmouth Ave, Kingston, ON K7M 1V5 Canada

**Keywords:** Indoor, Radon, Risk, Perception, Behaviour change models

## Abstract

**Background:**

Despite being the leading cause of lung cancer for non-smokers, few Canadians take action to test for and mitigate radon. This study’s aim was twofold: (1) to investigate predictors of radon testing and mitigation using the Precaution Adoption Process Model (PAPM) and Health Belief Model (HBM); and (2) to assess the impact on beliefs of receiving radon results above health guidelines.

**Methods:**

A convenience sample within Southeastern Ontario households was recruited to test their homes for radon (*N* = 1,566) for a pre-post quasi-experimental study. Prior to testing, participants were surveyed on risk factors and HBM constructs. All participants whose homes tested above the World Health Organization’s radon guideline (*N* = 527) were surveyed after receiving their results and followed for up to 2 years after. Participants were classified into PAPM stages and regression analyses were conducted to determine predictors between different stages (from deciding to test onwards). Paired bivariate analyses compared responses before and after receiving results.

**Results:**

Perceived benefits from mitigating was associated with progressing through all stages in the study’s scope. Perceived susceptibility to and severity of illness and perceptions of cost and time to mitigate were associated with progression through some PAPM stages. Homes with smokers or individuals under 18 were associated with not progressing through some stages. Home radon level was associated with mitigation. Attitudes towards many HBM constructs significantly decreased after receiving a high radon result.

**Conclusions:**

Public health interventions should target specific radon beliefs and stages to ensure households test and mitigate for radon.

## Background

Radon, a known carcinogen, is present in all homes, but it is both odourless and invisible [1]. Exposure to indoor radon is the leading cause of lung cancer for non-smokers [2–5]. There is no threshold for carcinogenic radon exposure [2], with lung cancer risk directly linked to concentration and length of exposure [6]. Frequently, international and national health organizations utilize different concentrations in their radon guidelines. For example, the World Health Organization’s (WHO) radon limit for residential dwellings is 100 Bq/m^3^ [6], the United States’ guideline is around 150 Bq/m^3^ [4], and Health Canada recommends mitigation at levels of 200 Bq/m^3^ or greater [2]. More specifically, if exposure is between 200–600 Bq/m^3^ mitigation within two years is advised by Health Canada, while for exposure above 600 Bq/m3, mitigation within one year is advised [2]. In Ontario, Canada, it is estimated that 91 lung cancer deaths would be prevented each year if all homes above 200 Bq/m^3^ were remediated and 233 per year if all homes above 100 Bq/m^3^ were [7].

Like many countries in the world, homeowners in Canada are responsible for radon testing and mitigation [2] and have demonstrated low awareness and action. In 2017, 52% of (non-apartment) Canadian households had heard of and 7% had tested for radon [8]. One recent study found only 12% of participants had tested their home and 3% had mitigated [9]. It is evident that public health interventions are required to increase radon testing and mitigation. These interventions will have the greatest likelihood of success if they can be targeted to specific factors that lead homeowners to test and mitigate [9]. There is limited research in Canada on these factors, especially in terms of behaviours and opinions towards mitigation after receiving a radon test result above health guidelines.

**Fig. 1:**
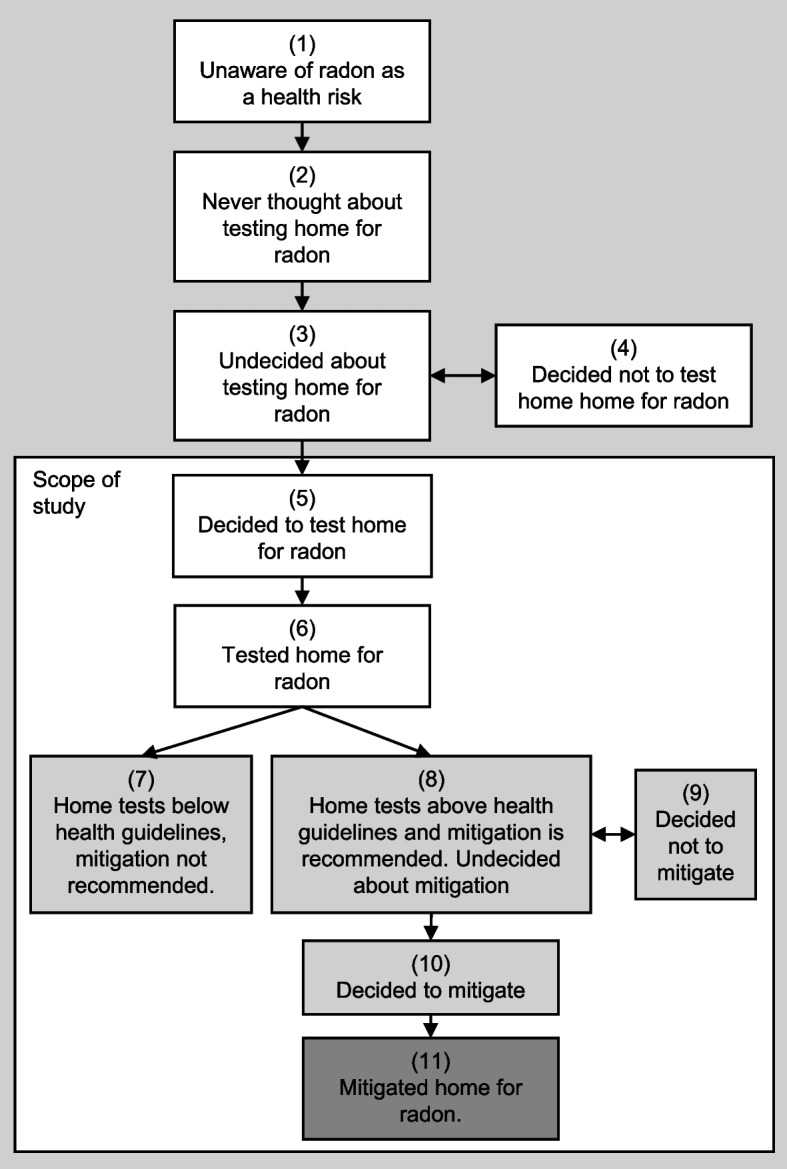
Hypothesized PAPM model for radon testing and mitigation, with the scope of the study highlighted. Legend: Testing phase in shown in white, the pre-mitigation phase in light grey, and the follow up phase in dark grey

Increased understanding of radon testing and mitigation is possible through using theoretical models of behaviour change [5, 9–11]. As radon is an environmental hazard with low awareness, a stage-based model that includes being unaware of the risk, such as the Precaution Adoption Process Model (PAPM), can be used to understand homeowners’ progression towards testing and mitigation. The PAPM has been used in the context of radon before [10, 11] and the radon specific PAPM stages hypothesized are shown in Fig. 1. While useful in understanding progress, the PAPM does not provide a framework for understanding what predicts progress through the stages. Instead, the Health Belief Model (HBM), a model for health concerns based on six constructs (perceived susceptibility, severity, barriers, and benefits, self-efficacy and cues to action) shown specifically for radon in Table 1, can be used. Previous international studies have assessed radon testing in terms of these models (or some of their stages/constructs) [10, 12–14], including a few examples of when they have been used in conjunction [11, 15].

**Table 1:** Health Beliefs Model (HBM) constructs related to radon

HBM construct	Radon testing- and/or mitigation-specific construct
Perceived susceptibility	To having high levels of radon in one’s area or home
	To illness from radon
Perceived severity	Of illness due to radon
Perceived barriers	To testing for radon
	To mitigating homes with high levels of radon
Perceived benefits	Of mitigating homes with high levels of radon
Self-efficacy	In testing one’s home for radon
	In mitigating ones’ home for radon
Cue to action^a^	To radon testing^a^
	To mitigation^a^

^a^Not included in the study

This study expands upon the literature’s radon application of the PAPM [10, 11] and HBM [11, 13, 15] by assessing the combined models from the deciding to test stage (Stage 5) through to the mitigation stage (Stage 11). The purpose of this study was to use these models as conceptual frameworks to understand factors influencing homeowners’ decisions to test and mitigate, and to guide future public health interventions. Conducted by the Kingston, Frontenac, and Lennox & Addington (KFL&A) region’s local public health agency (LPHA) from winter 2018/2019 through spring 2022, the project provided participants with radon tests and results, and then followed them for up to 24 months after to determine if action was taken to lower levels after receiving high results. Specifically, the objectives of this study were to: (1) investigate predictors of progress (including the HBM constructs) towards radon testing, intention to mitigate, and mitigation; and (2) assess the impact of receiving a radon result above health guidelines on HBM constructs.

## Methods

### Recruitment and data collection

A convenience sample of KFL&A homeowners was recruited through print and online media for the pre-post quasi-experimental study. As this study was part of a public health intervention by a LPHA, all interested homeowners were included and random sampling was not possible. Homeowners were excluded if they intended to sell or renovate within six months as this would affect their long-term follow-up in the study. They were also excluded if their home was used for business purposes (due to legal requirements on the LPHA) or if it did not have either a ground floor or basement (as the source of most radon in houses is in the soil on which the house is standing, therefore higher radon levels are more likely to exist on the lower levels of a home) [16].

Participants first completed an online survey (the pre-test survey) and if eligible were then contacted to pick up a radon test (AccuStar AlphaTrack AT-100 long term test) from a public health office during regular or extended hours. After 91 days of testing, participants returned their test to the office. Frequent reminders were sent to all participants throughout this period. The LPHA provided each participant with their result including interpretation based on public health guidelines.^1^ Five to seven weeks after receiving their result, participants whose homes tested above WHO health guidelines (100 Bq/m^3^) were invited by email to complete a post-test survey. These participants were also invited by email to complete supplementary follow-up surveys (6, 12 and 24 months) post-receiving their radon result; when participants indicated they had taken action to lower radon levels they were not included in further follow-up surveys. CheckMarket was used for all online surveys.

### Survey instrument

The behavioural models informed instrument design for the pre- and post-test surveys. Questions were designed based on HBM constructs and asked on a 7-point Likert scale. Many questions and response categories were adapted from surveys found in the literature [2, 12, 14, 17]. The pre-test survey contained questions related to both testing and mitigation, while the post-test survey focused on mitigation attitudes. Additionally, both main surveys asked participants if they intended to mitigate their homes. Some items conceptually required a home to have high levels of radon (e.g., “I wouldn’t have time to fix [radon] in my home.”). On the pre-test survey, this was phrased as a hypothetical (“if I had high levels of radon in my home”) which was switched to a factual statement in the post-test survey (“the high levels of radon in my home”). Additionally, based on radon risk factors, the pre-test survey asked about the smoking status and age of the youngest resident, and hours spent on the lower floors of the home. The follow-up surveys asked if participants had taken action to reduce radon levels in their homes. Open-ended questions were included in all surveys to elicit additional contextual information. For example, on the pre-test and post-test surveys, participants who believed that if their home had radon, it was likely that someone would get sick from it (based on the close-ended question) were then asked “Why do you think that it is likely someone will get sick from the radon in your home?”. On the post-test, participants were asked if there were other reasons why they would not fix their home’s high level of radon and if there was anything that would help them fix it.

### Data analysis

Participants were first classified into PAPM testing stages. All participants who returned a radon test were classified as Stage 6 (Tested), with the remaining pre-test survey participants classified as Stage 5 (Decided to Test). Participants whose homes tested above 100 Bq/m^3^ were then classified into a mitigation-specific stage based on their response to the mitigation intention question in the post-test survey. Participants who disagreed with the question “I plan to fix the high levels of radon in my home within two years” were classified as Stage 9 (Decided not to Mitigate), neutral participants as Stage 8 (Undecided about Mitigating), and participants who agreed as Stage 10 (Decided to Mitigate). For the final analysis, participants were classified into Stage 11 (Mitigated) if they had responded yes to taking action to lower radon levels on any of the three follow-up surveys.

Regression techniques were used to answer objective one (predictors of progress through the PAPM). Given the different stages captured in the study, three analyses were performed: (A) a testing-specific comparison between Stages 5 (Decided to Test) and 6 (Tested) using logistic regression; (B) a mitigation intention-specific comparison between Stages 8 (Undecided about Mitigating), 9 (Decided not to Mitigate) and 10 (Decided to mitigate) using multinomial logistic regression; and (C) a mitigation behaviour-specific comparison between Stage 11 (Mitigated) and those remaining in Stages 8–10 (Undecided, Decided not to, Decided to Mitigate) using logistic regression. All HBM constructs (from the pre-test survey for (A) and from the post-test survey for (B) and (C)) and risk factors were considered as potential predictor variables. Radon level (log-transformed) was also included in (B) and (C). Before the regression analyses, bivariate analysis (χ^2^ tests) were performed between each potential predictor variable and the outcome. Each predictor variable was dichotomized to increase power. Statistical testing between the variables determined the dichotomy as no apriori assumptions were applied; hence, different dichotomies could be used across variables and analyses. Only potential predictor variables significant at a cut-off of 0.1 were included in the regression models. Furthermore, a stepwise approach was used where only variables significant at a cut-off of 0.05 in the full model were included in the refined model.

Objective 2 was investigated using paired t-tests on the HBM questions asked in both the pre- and post-test surveys. Furthermore, this analysis was stratified by radon guideline (above the WHO guideline but below Health Canada’s, and above Health Canada) to determine any impact of radon level on changes observed.

All quantitative analyses were performed in R version 4.1.1 (The R Foundation for Statistical Computing). The open-ended data was categorized into major themes and sub-themes using NVivo 12 (QSR International). Key high level themes, including demonstrative quotations, from the post-test survey are provided as supplemental contextual information.

## Results

### Participants

A total of 1,566 eligible participants consented to participate, with 1,046 testing their homes for radon (Stage 6) and the remaining 520 participants staying in Stage 5 (Decided to Test). A summary of the characteristics of these participants is available in previously published work [18]. Notably, of the participants who picked up a test kit (1,118), the response rate was 93.7%. After testing, there were 527 (50.4%) participants whose homes tested above the WHO guidelines. Of these, 388 of them completed at least some of the post-test survey (response rate of 73.6%). Some differences existed between respondents and non-respondents to the post-test survey. As compared to respondents, non-respondents were more likely to believe they wouldn’t have time to mitigate and that their house would be worth less even after mitigating. Based on their responses to the intention to mitigate question, 85 participants (21.9%) were classified as Stage 8 (Undecided about Mitigating), 72 (18.6%) participants as Stage 9 (Decided not to Mitigate), and 227 (58.5%) as Stage 10 (Decided to Mitigate).

Of those that responded to the post-test survey, 370 (95.3%) responded to at least one follow-up survey and, of these, 323 (87.2%) responded until they had mitigated or at least to the last survey. Of those who responded to at least one, 176 (47.5%) indicated that they had taken action to lower radon levels (Stage 11).

### Predictors for progressing to testing home

Table 2 shows the potential predictor variables for progressing from Stage 5 (Decided to Test) to Stage 6 (Tested) and their bivariate association with the outcome. Ten variables were significant with a cut-off of 0.1: two risk factors, five HBM construct variables and three specific perceived barriers/self-efficacy questions. Across the Likert scale questions differing behaviours of the neutral group were observed; the table includes the specific dichotomy for each variable.

**Table 2:** χ^2^ results for testing-specific analysis (Stage 5 to 6)

Construct	Question (levels compared, N)	Stage 5 (Decided to Test) vs. Stage 6 (Tested) (χ^2^)
Demographic/ lifestyle variables	Age of the youngest person in a home (18 or under vs. older than 18, *N* = 1548)	40.29^***^
	Smoking status (any known current smoker in home vs. rest, *N* = 1566)	6.62^**^
	Hours spent on the lowest floor of home (4 levels, *N* = 1548)	1.95
	Hours spent in basement (5 levels, *N* = 1549)	1.46
Perceived susceptibility	Radon is a problem in my area/neighbourhood. (agree vs. disagree/neutral, *N* = 1513)	3.98^*^
	My home likely has enough radon that I should do something about it. (agree vs. disagree/neutral, *N* = 1514)	5.77
	If radon is in my home, it is likely that someone will get sick from it. (agree/neutral vs. disagree, *N* = 1518)	8.48^**^
Perceived severity	If someone in my household got sick from radon, it would be very serious. (agree vs. disagree/neutral, *N* = 1507)	14.23^***^
Perceived benefits	If I reduced the levels of radon in my home, it would reduce the chances of someone getting sick from it. (agree vs. disagree/neutral, *N* = 1491)	24.52^***^
Perceived barriers/self-efficacy	I do not know where to buy a radon test kit. (agree vs. disagree/neutral, *N* = 1488)	1.744
	If I did have a radon testing kit, I might make a mistake when testing my home for radon. (agree/neutral vs. disagree, *N* = 1493)	0.12
	The results of the radon tests are not reliable. (agree vs. disagree/neutral, *N* = 1495)	0.79
	I don’t trust companies that deal with radon. (agree vs. disagree/neutral, *N* = 1492)	1.19
	If I did test my home for radon and the test revealed high levels, I would not know how to find an experienced contractor to fix the problem. (agree/neutral vs. disagree, *N* = 1493)	2.22
	If I had high levels of radon in my home, I wouldn’t have the time to fix it. (agree/neutral vs. disagree, *N* = 1491)	9.75^**^
	If I had high levels of radon in my home, it would be too expensive to fix. (agree vs. disagree/neutral, *N* = 1493)	18.57^***^
	Even if a radon problem was fixed, my home would still be worth a lot less. (agree/neutral vs. disagree, *N* = 1494)	6.81^*^

Statistical significance level in the results denoted by: ‘***’ for < 0.001, ‘**’ for < 0.01, and ‘*’ for < 0.05

When these ten potential predictors were used in a logistic regression (*N* = 1,417), four variables were significant at a cut-off of 0.05 and were included in the refined regression model (*N* = 1,449); the results for the full and refined models can be seen in Table 3, including odds ratios. Having children or current smokers in the house decreased the odds of progressing to testing as did believing it would be too expensive to fix high levels of radon. Conversely, participants who perceived the benefits of mitigating were more likely to test.

**Table 3:** Logistic regression model results for testing-specific analysis (Stage 5 to 6)

Construct	Question	Full Model Odds Ratio (95% Confidence Interval) *N* = 1417	Refined Model Odds Ratio (95% Confidence Interval) *N* = 1449
Demographic/ lifestyle	Age of youngest person in home (older than 18 vs. 18 and under)	1.88 (1.47—2.39)^***^	1.85 (1.45 – 2.34)^***^
	Smoking status (rest vs. any known current smoker)	1.65 (1.11—2.45)^*^	1.66 (1.11 – 2.45)^*^
Perceived susceptibility	Radon is a problem in my area/neighbourhood. (agree vs. disagree/neutral)	1.50 (0.85—2.80)	
	My home likely has enough radon that I should do something about it. (agree vs. disagree/neutral)	1.66 (0.98—2.94)	
	If radon is in my home, it is likely that someone in my household will get sick from it. (agree vs. disagree/neutral)	1.32 (0.78—1.67)	
Perceived severity	If someone in my household got sick from radon, it would be very serious. (agree vs. disagree/neutral)	1.14 (0.74—2.29)	
Perceived benefits	If I reduced the levels of radon in my home, it would reduce the chances of someone getting sick from it. (agree vs. disagree/neutral)	1.57 (1.08—2.29)^*^	1.91 (1.39 – 2.62)^***^
Perceived barriers/self-efficacy	If I had high levels of radon in my home, I wouldn’t have the time to fix it. (agree/neutral vs. disagree)	0.78 (0.58—1.05)	
	If I had high levels of radon in my home, it would be too expensive to fix. (agree vs. disagree/neutral)	0.72 (0.52—1.00)^*^	0.64 (0.47 – 0.87)^**^
	Even if a radon problem was fixed, my home would still be worth a lot less. (agree/neutral vs. disagree)	0.82 (0.64—1.05)	

Statistical significance level in the models denoted by: ‘***’ for < 0.001, ‘**’ for < 0.01, and ‘*’ for < 0.05

### Predictors for progressing to deciding to mitigate home

There were 14 variables considered as potential predictors for progressing to Stage 10 (Decided to Mitigate) compared to Stages 8 (Undecided about Mitigating) or 9 (Decided not to Mitigate); these are shown in Table 4 with the bivariate analysis results. All but three of them were statistically significant at a cut-off of 0.1, specifically one risk factor, all the HBM constructs and specific barriers/self-efficacy questions, and the log-transformed radon level. Like with the previous modelling, differing behaviours were observed for the neutral group in Likert scale questions with the final dichotomy included in the table.

**Table 4:** χ2/ANOVA results for deciding to mitigate intention analysis (Stages 8, 9 and 10)

**Construct**	**Question (levels compared,** ***N***)	**Stage 8 (Undecided about mitigating) vs. Stage 9 (Decided not to) vs. Stage 10 (Decided to) (χ**^**2**^**)**
Demographic/ lifestyle variables	Age of youngest person in home (18 or under vs. 19 and over, *N*=384)	4.15
	Smoking status (only never smokers in home vs. rest, *N*=384)	9.18*
	Hours spent on the lowest floor of home (4 levels, *N*=383)	2.24
	Hours spent in basement (5 levels, *N*=383)	2.08
Perceived susceptibility	It is likely that someone in my household will get sick from radon. (agree/neutral vs. disagree, *N*=378)	53.04***
Perceived severity	If someone in my household got sick from radon, it would be very serious. (agree vs. disagree/neutral, *N*=379)	32.18***
Perceived benefits	If I reduced the levels of radon in my home, it would reduce the chances of someone getting sick from it. (agree vs. disagree/neutral, *N*=373)	106.1***
Perceived barriers/self-efficacy	The results of the radon tests are not reliable. (agree/neutral vs. disagree, *N*=383)	11.88**
	I don’t trust companies that deal with radon. (agree/neutral vs. disagree, *N*=383)	7.21*
	I don’t know how to find an experience radon contractor to fix the high levels of radon in my home. (agree/neutral vs. disagree, *N*=383)	9.73**
	I don’t have time to fix the high levels of radon in my home. (agree/neutral vs. disagree, *N*=383)	35.76***
	It is too expensive to fix the high levels of radon in my home (agree vs. disagree/neutral, *N*=383)	19.59***
	My home is worth a lot less because of the high levels of radon, even if I fix it. (agree/neutral vs. disagree, *N*=384)	5.73†
Radon level	Log-transformed (ANOVA result, *N*=384)	***

Statistical significance level in the results denoted by: ‘***’ for < 0.001, ‘**’ for < 0.01, ‘*’ for < 0.05 and † for <0.1

In the full multinomial logistic regression model containing the 11 variables (*N* = 367), six variables were significant at a cut-off of 0.05 for predicting Stage 9 (Decided not to) or 10 (Decided to) (compared to Stage 8 (Undecided)) and were included in the refined model (Table 5). In the final model, different variables were associated with an outcome of Stage 9 (Decided not to) versus Stage 10 (Decided to). Participants who did not perceive susceptibility to illness or benefits from mitigating were more likely to decide not to. Never smoking households, increasing radon levels, perceiving the severity of illness and the benefits of mitigating were all associated with increased odds in intention to mitigate. Conversely, participants who agreed with/were neutral to the statement “I don’t have time to fix the high levels of radon in my home” were less likely to decide to mitigate.

**Table 5:** Multinomial logistic regression model results for mitigation intention analysis (Stages 8, 9 and 10)

**Construct**	**Question**	**Full Model Odds Ratio (95% Confidence Interval) *N*** **= 367**		**Refined Model Odds Ratio (95% Confidence Interval) *N*** **= 370**	
		**Decided not to (Stage 9)**	**Decided to (Stage 10)**	**Decided not to (Stage 9)**	**Decided to (Stage 10)**
Demographic/lifestyle	Smoking status (ever/former/ unsure vs. never smoker)	0.51 (0.24 – 1.06)	0.48 (0.26 – 0.89)*	0.55 (0.27 – 1.14)	0.53 (0.29 – 0.95)*
Perceived susceptibility	It is likely that someone in my household will get sick from radon. (agree/neutral vs. disagree)	0.27 (0.12 – 0.59)**	0.99 (0.52 – 1.87)	0.25 (0.12 – 0.56)***	0.95 (0.52 – 1.76)
Perceived severity	If someone in my household got sick from radon, it would be very serious. (agree vs. disagree/neutral)	0.98 (0.44 – 2.17)	2.33 (1.10 – 4.92)*	0.97 (0.45 – 2.09)	2.44 (1.18 – 5.05)*
Perceived benefits	If I reduced the levels of radon in my home, it would reduce the chances of someone getting sick from it. (agree vs. disagree/neutral)	0.26 (0.11 – 0.61)**	2.87 (1.40 – 5.89)**	0.27 (0.12 – 0.59)**	2.72 (1.38 – 5.36)**
Perceived barriers/self-efficacy	The results of the radon tests are not reliable. (agree/neutral vs. disagree)	0.63 (0.28 – 1.45)	0.58 (0.29 – 1.16)		
	I don’t trust companies that deal with radon. (agree/neutral vs. disagree)	1.07 (0.44 – 2.60)	1.13 (0.55 – 2.33)		
	I don’t know how to find an experience radon contractor to fix the high levels of radon in my home. (agree/neutral vs. disagree)	1.67 (0.72 – 3.88)	1.89 (0.93 – 3.84)		
	I don’t have time to fix the high levels of radon in my home. (agree/neutral vs. disagree)	0.73 (0.31 – 1.71)	0.29 (0.15 – 0.58)***	0.83 (0.40 – 1.73)	0.29 (0.16 – 0.52)***
	It is too expensive to fix the high levels of radon in my home (agree vs. disagree/neutral)†	0.92 (0.29 – 2.94)	0.49 (0.19 – 1.23)		
	My home is worth a lot less because of the high levels of radon, even if I fix it. (agree/neutral vs. disagree)	0.77 (0.36 – 1.65)	0.60 (0.32 – 1.14)		
Radon level	Log-transformed	0.49 (0.17 – 1.43)	3.79 (1.89 – 7.57)***	0.52 (0.18 – 1.50)	3.98 (2.00 – 7.91)***

Statistical significance level in the models denoted by: ‘***’ for < 0.001, ‘**’ for < 0.01, and ‘*’ for < 0.05

### Predictors for progressing to mitigating home

Table 6 shows the 14 variables considered as potential predictors for progressing to Stage 11 (Mitigated) (compared to remaining in Stages 8–10 combined (Undecided/Decided not to/Decided to Mitigate)). Using a cut-off of 0.1, 8 were significant: 3 HBM construct questions, 4 specific barrier/self-efficacy questions and the log-transformed home radon level. As before, different dichotomizations were observed for Likert scale questions.

**Table 6:** χ2/ANOVA results for mitigation behaviour analysis (Stages 8-10 to 11)

**Construct**	**Question (levels compared,** *N***)**	**Not mitigated (Stages 8-10) vs. mitigated (Stage 11) (χ**^**2**^**)**
Demographic/ lifestyle variables	Age of youngest person in home (18 or under vs. 19 and over, *N*=370)	0.16
	Smoking status (only never smokers in home vs. rest, *N*=370)	1.61
	Hours spent on the lowest floor of home (4 levels, *N*=370)	3.68
	Hours spent in basement (5 levels, *N*=383)	3.16
Perceived susceptibility	It is likely that someone in my household will get sick from radon. (agree/neutral vs. disagree, *N*=366)	8.28**
Perceived severity	If someone in my household got sick from radon, it would be very serious. (agree vs. disagree/neutral, *N*=362)	3.30†
Perceived benefits	If I reduced the levels of radon in my home, it would reduce the chances of someone getting sick from it. (agree vs. disagree/neutral, *N*=364)	20.14***
Perceived barriers/self-efficacy	The results of the radon tests are not reliable. (agree/neutral vs. disagree, *N*=363)	2.60
	I don’t trust companies that deal with radon. (agree/neutral vs. disagree, *N*=354)	6.93**
	I don’t know how to find an experience radon contractor to fix the high levels of radon in my home. (agree/neutral vs. disagree, *N*=362)	4.68*
	I don’t have time to fix the high levels of radon in my home. (agree/neutral vs. disagree, *N*=364)	28.07***
	It is too expensive to fix the high levels of radon in my home (agree/neutral vs. disagree, *N*=364)	18.66***
	My home is worth a lot less because of the high levels of radon, even if I fix it. (agree/neutral vs. disagree, *N*=362)	1.19
Radon level	Log-transformed (ANOVA result, *N*=370)	***

Statistical significance level in the results denoted by: ‘***’ for < 0.001, ‘**’ for < 0.01, ‘*’ for < 0.05 and † for <0.1

The full logistic regression (*N* = 353) showed 5 variables significant at a 0.05 cut-off for inclusion in the refined model (*N* = 360) (Table 7). Increasing radon level and perceiving benefits of mitigating were associated with increased odds of mitigation while believing that they didn’t have time or that it was too expensive were associated with decreased odds.

**Table 7:** Logistic regression model results for mitigation behaviour analysis (Stages 8-10 to 11)

**Construct**	**Question**	**Full Model Odds Ratio (95% Confidence Interval) *N*=353**	**Refined Model Odds Ratio (95% Confidence Interval) *N*=360**
Perceived susceptibility	It is likely that someone in my household will get sick from radon. (agree/neutral vs. disagree)	1.11 (0.66 - 1.86)	
Perceived severity	If someone in my household got sick from radon, it would be very serious. (agree vs. disagree/neutral)	0.93 (0.50 - 1.73)	
Perceived benefits	If I reduced the levels of radon in my home, it would reduce the chances of someone getting sick from it. (agree vs. disagree/neutral)	1.86 (1.05 - 3.33)*	1.93 (1.15 – 3.28)*
Perceived barriers/ self-efficacy	I don’t trust companies that deal with radon. (agree/neutral vs. disagree)	0.65 (0.39 - 1.07)*	0.69 (0.43 – 1.12)
	I don’t know how to find an experience radon contractor to fix the high levels of radon in my home. (agree/neutral vs. disagree)	1.32 (0.76 - 2.31)	
	I don’t have time to fix the high levels of radon in my home. (agree/neutral vs. disagree)	0.40 (0.24 – 0.69)***	0.43 (0.26 – 0.71)**
	It is too expensive to fix the high levels of radon in my home (agree/neutral vs. disagree)	0.53 (0.28 - 0.97)*	0.52 (0.28 – 0.95)*
Radon level	Log-transformed	3.52 (2.16 – 5.92)***	3.52 (2.19 – 5.83)***

Statistical significance level in the models denoted by: ‘***’ for < 0.001, ‘**’ for < 0.01, and ‘*’ for < 0.05

### Impact of receiving a high radon result

Table 8 shows the paired t-test results from comparing HBM constructs before and after receiving radon results above health guidelines (overall and stratified by radon level). Overall, perceived susceptibility to and severity from illness, perceived benefits, and three specific perceived barriers/self-efficacy questions (time and cost to remediate and reliability of the tests) showed a statistically significant decrease between the pre- and post-test surveys with the greatest numerical difference in perceived susceptibility. Perceptions towards knowing how to find a radon contractor increased statistically, which was information provided to participants alongside their test result. Stratified results were comparable to the overall results, except that differences in the belief in the reliability of tests were not significant in the higher radon level group.

**Table 8:** Differences in perceptions towards radon mitigation before and after receiving a radon test result

**Construct**	**Question**	**All participants**		**Between 100 to 200 Bq/m**^**3**^		**Above 200 Bq/m**^**3**^	
		Percentage difference in agreeing with the construct/ barrier (n/N)	Mean change (t-score)	Percentage difference in agreeing with the construct/ barrier (n/N)	Mean change (t-score)	Percentage difference in agreeing with the construct/ barrier (n/N)	Mean change (t-score)
Perceived susceptibility	It is likely that someone in my household will get sick from radon.^a^	-48.68% (184/378)	1.77 (19.61***)	-56.95% (127/223)	2.14 (18.63***)	-36.77% (57/155)	1.23 (9.18***)
Perceived benefits	If I reduced the levels of radon in my home, it would reduce the chances of someone getting sick from it.	-23.12%(86/372)	0.82 (9.31***)	-32.26% (70/217)	1.14 (9.78***)	-10.32% (16/155)	0.37 (2.96**)
Perceived severity	If someone in my household got sick from radon, it would be very serious.	-10.40% (39/375)	0.47 (6.36***)	-13.12% (29/221)	0.56 (5.62***)	-6.49% (10/154)	0.34 (3.14**)
Perceived barriers/Self-efficacy	It is too expensive to fix the high levels of radon in my home.^a^	28.68% (109/380)	-1.25 (-3.37***)	24.55% (55/224)	-1.37 (-11.12***)	34.62% (54/156)	-1.08 (-7.55***)
	I don’t have the time to fix the high levels of radon in my home.^a^	9.23% (35/379)	-1.04 (-10.98***)	9.38% (21/224)	-1.19 (-9.45***)	9.03% (14/155)	-0.82 (-5.80***)
	The results of the radon tests are not reliable.	9.76% (37/379)	-0.256 (-2.65**)	8.04% (18/224)	-0.26 (-2.06*)	12.26% (19/155)	-0.25 (-1.65)
	My home is worth a lot less because of the high levels of radon, even if I fix it.^a^	-1.58% (6/379)	-0.04 (-0.48)	-1.79% (4/223)	-0.07 (-0.56)	-1.28% (2/156)	-0.01 (-0.08)
	I don’t trust companies that deal with radon.	2.89% (11/380)	-0.13 (-1.44)	5.36% (12/224)	-0.21 (-1.78)	-0.64% (1/156)	-0.01 (-0.05)
	I don’t know how to find an experienced radon contractor to fix the high levels of radon in my home.^a^	-21.96% (83/378)	0.95 (8.32***)	-23.21% (52/224)	0.96 (6.50*** )	-20.13% (31/154)	0.93 (5.18***)

^a^Indicates that the question change between the pre- and post-radon testing from a hypothetical scenario of radon being in one’s home (“If radon is in my home”) to the known radon level (“the radon in my home” or “the high levels of radon in my home”)

Statistical significance level in the models denoted by: ‘***’ for < 0.001, ‘**’ for < 0.01, and ‘*’ for < 0.05

### Key themes related to mitigation

Across the post-test survey open-ended questions, the most prominent theme was that participants perceived homes to not have high enough radon levels to need mitigation, with a total of 99 participants (25.5% of all respondents) providing at least one comment on this. This response was more prevalent in those whose homes tested between 100–200 Bq/m^3^, but was observed across all radon levels. Furthermore, respondents stated that the existence of multiple guidelines meant that the risk of illness between 100–200 Bq/m^3^ is low. Examples of this theme are found below:

“I have NO radon in my home”

“First of all, the test showed 185, below Health Canada's guideline of 200, so although it would be nice to meet the more stringent WHO guideline, I can't imagine that the findings are that serious.”

The most common reason provided by participants for why they would not fix their home’s radon levels was financial – both in terms of the mitigation costs and the impact on resale value.

“the drop in home value when you install active mitigation is a scary thing!”

“I don't believe the risk is worth the expense”

“We are on a fixed income and need to plan for expenditures. … Radon will have to wait but it is on the to-do list.”

When asked if there was anything that would help them mitigate, 115 respondents (43.9%) mentioned government financial assistance (e.g., subsidies/grants or tax cuts/rebates).

“A government grant or tax cut for retrograde installation would help dramatically.”

## Discussion

The objective of this study was to guide targeted future radon testing and mitigation public health intervention utilizing the PAPM and HBM. Specifically, the first objective was to identify factors which increase testing and mitigation. This study determined that believing there are benefits from mitigation predicted progress through all PAPM stages studied. Other factors (smokers and age of youngest resident, perceived susceptibility to and severity of illness, barriers of cost and time to mitigate, and home radon level) predicted progress through some but not all stages. This suggests that interventions focusing on the benefits, cost, and ease of mitigation, and also the severity and susceptibility of illness would have the greatest impact on increasing radon testing (amongst those already decided to test) and mitigation. These could include communication campaigns, and structural, public health programming and policy interventions improving access to and decreasing the cost of mitigation (e.g., tax rebates, financial assistance).

Overall, the testing-specific findings of this study are consistent with previous literature from other areas of the world, though the relative scale of the predictors vary [11, 13, 15, 19, 20]. One notable difference is that previous literature has placed a greater emphasis on perceived susceptibility and severity [11, 13, 15, 20], while this study emphasized perceived benefits of mitigation.

Recent literature examining radon testing and mitigation perceptions and behaviours is lacking. One cross-sectional study in Ottawa, Ontario, which used the Protection Motivation Theory, found perceived susceptibility, severity, and smoking (amongst other factors unmeasured by this study) associated with having tested and mitigated, but the sample size for those who had mitigated was small (under 20) [9]. Conversely, this pre-post quasi-experimental study found that perceived susceptibility was only associated with testing behaviours and being undecided regarding mitigating (as compared to deciding not to) and perceived severity with deciding to mitigate (compared to being undecided). Having smokers in the home was associated with not testing and deciding not to mitigate, which is concerning given their elevated risk of developing lung cancer due to the synergistic effect of cigarette smoking and radon [21], even at concentrations below recommended guidelines [22]. The Ottawa study did not quantitatively measure self-efficacy, barriers or perceived benefits, but did qualitatively observe that the cost of remediation was a barrier [9, 23]. Additionally, multiple studies have found that increasing radon levels would increase the odds of mitigating, a finding corroborated by this study [24–26].

One hypothesis in the literature is that different HBM constructs will be relevant in different stages of the PAPM [10]. This study confirmed that some constructs change while others (notably perceived benefits) remain constant at least through the PAPM stages investigated, including the previously unstudied mitigation-specific stages. Furthermore, all the HBM constructs showed statistically significant associations with progression through testing and mitigation. Together these findings suggest that using these combined models is appropriate and can provide a critical understanding of testing and mitigation behaviours.

Alongside the first objective, the study aimed to assess the impact of receiving a radon result above health guidelines. One of the major findings of this study is that participants became more negative towards radon testing and mitigation after receiving a radon result above guidelines. This presents a public health communication challenge as mitigation is required to reduce the health risk. It is insufficient to achieve testing, and so, interventions need to also focus on perceptions amongst those who have tested and initiatives to support mitigation. This is supported by a previous study that also identified the importance of focusing interventions on mitigation follow-through [26]. Those with higher radon levels were more likely to intend to mitigate and to mitigate, which means those at the highest risk for lung cancer are the most likely to take action to reduce the risk. One possible contributing factor to the impact of radon level is the use of two different guidelines (WHO versus Health Canada) and the accompanying messaging provided by the LPHA. Qualitative results indicated that many individuals who tested between 100–200 Bq/m^3^ did not believe that there was a risk at this level. This is concerning as the WHO reported that lung cancer risk increases 16% per 100 Bq/m^3^ increase in long-term radon levels [21]. If the WHO guideline was followed and all homes in Ontario above this guideline were lowered to background levels, 28% of radon-attributable lung cancer deaths could be prevented [7]. This suggests simplifying radon guidelines and messaging to be consistent with the WHO might increase understanding of the risk from radon at all levels. As an intermediary step, providing support in interpreting test results in the context of national and international guidelines could address homeowners in making mitigation decisions.

### Limitations

The study is limited in that it was a convenience sample of homeowners who self-selected into a study being conducted by the LPHA. As such, the participants may represent a more engaged and health-seeking audience, so there may be limits to the generalizability of this study. Methods were used during recruitment to minimize this (e.g., every household in the region received a postcard inviting them to participate and the study was well covered by local media). Additionally, this study observed extremely high response rates throughout which limits bias from within the population studied.

Radon testing and mitigation are household-level behaviours but the surveys were conducted on individuals and so the measured constructs may not reflect the whole household. Additionally, the follow-up surveys did not ask for specifics on what type of mitigation methods were used, notably if certified mitigation specialists were employed. Further work would be beneficial to understand the type of actions homeowners take when their homes test above health guidelines.

The two-year follow-up period included the COVID-19 pandemic and associated lockdowns. This may have affected access to conduct mitigation and/or the pandemic may have had financial impacts on the participants. Either of these could have resulted in decreased mitigation occurring.

This study was one component of larger project assessing radon risk and beliefs in the LPHA region. As part of this larger project, qualitative data revealed that the project, and the role of LPHA in it, might have increased the uptake of radon testing due to convenience and trust in the organization [18]. This could have implications for the results of the study and for future public health programming.

## Conclusions

This study used the PAPM and HBM to understand predictors of radon testing and mitigation behaviours. The study is unique in having used a pre-post quasi-experimental design to measure the impact of receiving radon results above health guidelines and to determine associations between perceptions and beliefs to achieving the health protective step of mitigation. Statistical analyses determined that perceiving the benefits from mitigation, perceiving the susceptibility to and severity from illness, barriers of cost and time to mitigate as well as not having smokers in the home and the radon level were associated with moving through different stages of the PAPM. Furthermore, the study revealed the negative impact to perceptions upon receiving a radon level above health guidelines.

The findings of this study will inform the development of specific messaging campaigns and public health programming targeting perceptions most likely to be the underlying reasons for not testing and not mitigating. Furthermore, it also supports changes in radon policies (e.g., financial support for mitigating, aligning national radon guidelines with the WHO guideline) to further address this health risk.

## Data Availability

Available from the corresponding author on reasonable request.

## Abbreviations

Bq/m^3^: Becquerels per metre cubed
HBM: Health Belief Model
KFL&A: Kingston, Frontenac, and Lennox & Addington
LPHA: Local Public Health Agency
PAPM: Precaution Adoption Process Model
WHO: World Health Organization

## Footnotes

1 Specifically, participants whose:

- home radon level tested above 600 Bq/m^3^ were recommended to take action to lower radon levels within one year,
- home radon level tested between 200 Bq/m^3^ and 600 Bq/m^3^ were recommended to take action to lower radon levels within two years; and.
- home radon level tested between 100 Bq/m^3^ and 200 Bq/m^3^ were recommended to consider taking action to lower radon levels.
